# Gut dysbiosis is linked to metabolic syndrome in obese Egyptian women: potential treatment by probiotics and high fiber diets regimen

**DOI:** 10.1038/s41598-024-54285-5

**Published:** 2024-03-05

**Authors:** Nayera E. Hassan, Sahar A. El-Masry, Salwa M. El Shebini, Nihad H. Ahmed, Fouad Mohamed T, Mohammed I. Mostafa, Mahmoud A. S. Afify, Ayat N. Kamal, Mai M. Badie, Adel Hashish, Khadija Alian

**Affiliations:** 1 Biological Anthropology Department, Medical Research and Clinical Studies Institute, National Research Centre, 33 El-Buhouth St., Dokki, Giza, 12622 Egypt; 2 Nutrition and Food Science Department, Food and Industries and Nutrition Research Institute, National Research Centre, Giza, Egypt; 3 Food and Dairy Microbiology Department, Food and Industries and Nutrition Research Institute, National Research Centre, Giza, Egypt; 4 Clinical Pathology Department, Medical Research and Clinical Studies Institute, National Research Centre, Giza, Egypt; 5 Children With Special Needs Department, Medical Research and Clinical Studies Institute, National Research Centre, Giza, Egypt

**Keywords:** Gut microbiota, Metabolic syndrome, Probiotics, High fiber diet, Nutrition, Weight management

## Abstract

Metabolic syndrome (MetS) is defined as a cluster of glucose intolerance, hypertension, dyslipidemia, and central obesity with insulin resistance. The role of gut microbiota in metabolic disorders is increasingly considered. To investigate the effects of probiotic supplements and hypocaloric high fiber regimen on MetS in obese Egyptian women. A longitudinal follow-up intervention study included 58 obese Egyptian women, with a mean age of 41.62 ± 10.70 years. They were grouped according to the criteria of MetS into 2 groups; 23 obese women with MetS and 35 ones without MetS. They followed a hypocaloric high fiber regimen weight loss program, light physical exercise, and received a probiotic supplement daily for 3 months. For each participating woman, blood pressure, anthropometric measurements, basal metabolic rate (BMR), dietary recalls, laboratory investigations, and microbiota analysis were acquired before and after 3 months of follow-up**.** After intervention by the probiotic and hypocaloric high fiber regimen and light exercise, reduction ranged from numerical to significant difference in the anthropometric parameters, blood pressure, and BMR was reported. All the biochemical parameters characterized by MetS decreased significantly at p ≤ 0.05–0.01. Before the intervention, results revealed abundant of Bacteroidetes bacteria over Firmicutes with a low Firmicutes/Bacteroidetes ratio. After the intervention, Log Lactobacillus, Log Bifidobacteria, and Log Bacteroidetes increased significantly in both groups, while Log Firmicutes and the Firmicutes/Bacteroidetes Ratio revealed a significant decrease. In conclusion, this study’s results highlight a positive trend of probiotics supplementation with hypocaloric high-fiber diets in amelioration of the criteria of the Mets in obese Egyptian women.

## Introduction

The metabolic syndrome (MetS) is a complex disorder produced by a number of interconnected factors that raises the risk of heart disease and type 2 diabetes. Obesity is the most common antecedent of metabolic syndrome, which can be targeted in the development of new treatments^1^.

MetS frequently coexists with an imbalance in the gut microbiota, which damages the gut barrier and causes a low-grade inflammatory response in the body. This leads to insulin resistance through metabolites that alter host metabolism and hormone release, creating a vicious cycle that encourages the progression of MetS^2^.

Obesity is a precursor to MetS, thus treating it with physical activities (exercises), behavioral adjustments (counseling), calorie-restricted diets, weight-loss medicines, and finally weight-loss surgery will be critical in managing and controlling MetS. Exercise and behavior modification, on the other hand, necessitate a high level of mental control and are difficult to implement. Young obese have also been proven to be less effective on calorie-restricted diets. Similarly, current pharmacological medications have limitations such as negative side effects and expensive treatment costs^3^.

Probiotic supplementation improves anthropometric parameters in overweight and obese patients with linked metabolic illnesses, according to the findings of a meta-analysis study of Perna et al.^4^.

Dietary options, such as creating natural food products with probiotics and prebiotics to modify MetS, will be a cost-effective solution with no risk of negative health consequences. Probiotics are live microorganisms having a generally recognized as safe (GRAS) status, when given in sufficient doses, provide a health benefit to the host^5^.

Ingestion of dietary fiber has been thought to reverse the metabolism dysfunction. Changes occur due to microbial fermentation followed by the production of short chain fatty acids (SCFAs) that improve glucose and lipid parameters^6^**.**

Targeting intestinal microbiota with synbiotic (probiotic dietary supplements containing prebiotic additives), could be a promising intervention in a comprehensive nutritional approach to reducing obesity. Weight loss from a low-carbohydrate, high-protein diet can be significant, but it can also have potential health consequences from increased bacterial fermentation of undigested protein in the colon and subsequent changes in the composition of the intestinal flora. Treating of obesity-induced by destruction of the gut microbiota- by symbiotic may be more effective than supplementation with probiotics alone, as it helps the growth and survival of positive bacteria^7^. Stojanov et al.^8^ stated that the two most important bacterial phyla in the gastrointestinal tract, Firmicutes and Bacteroidetes, have gained much attention in recent years.

Aim of this study was to investigate the effects of probiotic supplement and hypocaloric high fiber regimen on the metabolic syndrome in Egyptian obese women. The study focused on the effectiveness of this dietary therapy in inducing body weight loss, improving the related metabolic disorders and the dysbiosis.

## Subjects and methods

Fifty eight obese Egyptian women were included in a longitudinal follow up intervention study, with mean age 41.62 ± 10.70 years. It was carried out at the Medical Research Excellence Centre (MERC), National Research Centre. It was a part of a cross-sectional survey of a project funded by National Research Centre (NRC) Egypt, 2019–2022 entitled ‘‘Gut Microbiota in Obesity and Metabolic syndrome among obese women: Interactions of the Microbiome, Epigenetic, Nutrition and Probiotic Intervention” (12th Research Plan of the NRC). The cross-sectional survey included 82 obese women with age ranged from 25 up to 60 years. Fifty eight women only of them completed the longitudinal follow up intervention study.

The study protocol was conformed to the ethical guidelines of the 1975 Declaration of Helsinki and was approved by the Ethics Committee of the NRC (Approval no 19/236). All participants provided their informed consent.

The participants of the study were enrolled in weight loss program. Those enrolled were initially obese and had a mean BMI of 38.32 ± 4.01 kg/m^2^. They followed hypocaloric high fiber regimen weight loss program eating plan, physical exercise, additionally received a probiotic supplement daily for 3 months. Women with conditions that may impact gut microbiota (smoking, gastrointestinal, autoimmune, and metabolic diseases and medications, particularly antibiotics) were not included in the study.

### Methods

For each participated woman, blood pressure, anthropometric measurements, basal metabolic rate (BMR), dietary recalls, laboratory investigations and microbiota analysis were acquired before and after 3 months of hypocaloric high fiber regimen (as prebiotic) weight loss program eating plan, light physical exercise and probiotic supplement intervention.

#### Blood pressure

Blood pressure was measured using the standardized mercury sphygmomanometer with a suitable cuff size. It was measured on the left arm while the participated women were sitting relaxed for 5 min. Two readings were obtained and the average was recorded. Systolic blood pressure (SBP); determined by the onset of the “tapping” korotkoff sounds (K1), while the fifth korotkoff sound (K5), or the disappearance of korotk off sounds, as the definition of diastolic blood pressure (DBP) were recorded.

#### Anthropometric measurements

Body weight, height and waist circumference were measured, following the recommendations of the “International Biological Program”^9^. Body weight (BW) was determined to the nearest 0.01 kg using a Seca Scale Balance, with the woman wearing minimal clothes and with no shoes. Body height (Ht) was measured to the nearest 0.1 cm using a Holtain portable anthropometer. Waist circumference (MWC) was measured at the midpoint between the lower curvature of the last fixed rib and the superior curvature of the iliac crest, with the woman in an upright standing position and their arms alongside the body, feet together, and abdomen relaxed. Body Mass Index was calculated [BMI: weight (in kilograms) divided by height (in meters squared)]. The participated women were all chosen as obese; as their BMI ≥ 30 kg/m^2^.

#### Basal metabolic rate

It was assessed using the TANITA Body Composition Analyzer. As specified by the manufacturer (Tanita Body Composition Analyzer-MC-780 MA III), the unit was calibrated before testing. The participant stood on the foot board of the device, while she was holding the 2 handles carefully, each by one hand at the same time. By using her sex, age, weight and height, approximated to the nearest unit, the Basal Metabolic Rate (BMR in kilo calories: the rate at which the body uses energy, while at rest, to maintain vital functions such as breathing and keeping warm) was measured .

#### Dietary recalls

Information from each participant about her usual pattern of food intake was obtained. Data were collected by means of dietary interview consisting of 24 h recall that repeated for 3 days, and a food frequency questionnaire. The total dietary intake was analyzed using Computer program Nurisurvey for window—copyright^10^.

#### Blood sampling and laboratory investigations

In the morning, venous blood samples were drawn from the participated females, using venipuncture. Biochemical parameters were performed on sera that were stored at -70 C° until used for assessment of fasting blood glucose, insulin and lipid profile. All were done in the laboratory of “Medical Excellence Research Center MERC” which is a part of “NRC”, Egypt.

Fasting blood sugar (FBG) level was measured using the automated clinical chemistry analyzer Olympus AU 400 analyzer. ***Serum insulin*** was assessed using Enzyme Immunoassay Test Kit Catalog No. E29-072 **(Immunospec Corporation).** Then **insulin resistance (IR)** was calculated according to Mathews et al.^11^ using the following equation: ${\text{IR}}\, = \,{\text{fasting }}\;{\text{glucose }}\left( {{\text{mg}}/{\text{dl}}} \right) \, \times {\text{ fasting}}\;{\text{ insulin }}\left( {{{\upmu {\rm IU}}}/{\text{ml}}} \right)/{4}0{5}$.

Serum levels of total cholesterol (TC), triglycerides (TG), high density lipoprotein cholesterol (HDL-C) were measured by standardized enzymatic procedures, using kits supplied by Roche Diagnostics (Mannheim, Germany) on the Olympus AU 400 automated clinical chemistry analyzer. Low density lipoprotein cholesterol (LDL-C) was calculated according to formula of Friedewald et al.^12^ as follows: ${\text{LDL - C}}\, = \,{\text{Total cholesterol}} - {\text{Triglycerides}}/{5}\, + \,{\text{HDL - C}}$ .

Clinically, a patient is considered to have MetS when three or more of the following five conditions exist, which are (1) waist circumference ≥ 88 cm in women, (2) blood pressure ≥ 135/85 mmHg, (3) triglycerides ≥ 150 mg/dl, (4) HDL-C < 50 mg/dl in women, and (5) fasting glucose ≥ 100 mg/dl^13^.

#### Microbiota analysis

To characterize effects of the weight loss program eating plan, light physical exercise and probiotic supplement on gut microbiota of the study participants, fecal samples were obtained before and after the intervention, gene sequence analysis was performed, and individual variations of gut microbiota were compared.

The proportion of Lactobacillus and Bifidobacteria; and Firmicutes/Bacteroidetes ratio strains were assessed in the stool of all participants by using the real time PCR (Polymerase Chain Reaction). Specimen collection and preparation: Stool was collected by defecation in a plain sterilized container allowed to be frozen. Specimen Storage and Preparation: stool was frozen on at − 20°. The primers and probes were used to detect *Bifidobacterium* spp. and *Lactobacillus* spp.; and *Firmicutes* spp. and *Bacteroidetes* spp., where based on 16S rRNA gene sequences retrieved from the National Center for Biotechnology Information databases by means of the Entrez program^14^*.* Reagents provided by kits: DNA extraction Kit. Assay procedure: DNA extraction: The QIAamp DNA Stool Minikit (Qiagen) was used to extract DNA from one gram of fresh or frozen stool sample according to the manufacturer's instructions. Bacterial quantification by real-time PCR was done.

### Intervention phase

All the participants were provided with the weight loss program eating plan, light physical exercise and probiotic supplement for 3 months:

1. Dietary modification plan was followed; under the supervision of a dietary consultant; by using different regimens aiming to correct the wrong food habits and to supply patients with the deficient nutrients (hypo caloric high fiber regimen). Through recognize and identify the association between food intake and dietary pattern among the studied groups. Dietary therapy was done in the form of different dietary regimens which could supply the requirement nutrient intake, and was followed to assess the impact of a dietary behavior modification intervention to reach the ideal weight for age and sex. Nutritional education and behavior modification were done through: pre-participation evaluation, designing the program and patient education, specific dietary programs for each age group; and weekly classes for follow up. The typical daily dietary meal plain regimen followed for 3 months was presented in Table 3.
2. Performing light aerobic exercise in the form of walking for 30 min/5 days/week or for 150 min/week.
3. In addition, they were provided with probiotic supplementation: 100 g from dietary supplement product (fermented milk in form Yogurt) which containing probiotic strains (10^8^/g). It was taken orally once a day for 3 months. It was distributed weekly. Probiotics strains were obtained from a capsule of probiotic “GNC ultra probiotic complex 100” which contains a mix of probiotic strains which used as it was isolated by National Research Centre (NRC) probiotics lab.

#### Principle of test

- The proportion of Lactobacillus and Bifidobacteria; and Firmicutes/Bacteroidetes ratio strains were assessed in the stool of all participants by using the real time PCR (Polymerase Chain Reaction).
-Specimen collection and preparation: Stool was collected by defecation in a plain sterilized container allowed to be frozen.
-Specimen Storage and Preparation: stool was frozen on at − 20°. The primers and probes were used to detect *Bifidobacterium* spp. and *Lactobacillus* spp.; and *Firmicutes* spp. and *Bacteroidetes* spp., where based on 16S rRNA gene sequences retrieved from the National Center for Biotechnology Information databases by means of the Entrez program^14^.
-Reagents provided by kits: DNA extraction Kit
-Assay procedure: DNA extraction: The QIAamp DNA Stool Minikit (Qiagen) was used to extract DNA from one gram of fresh or frozen stool sample according to the manufacturer's instructions.
  - Bacterial quantification by real-time PCR

#### Follow-up screening phase

All the participated women were followed weekly to measure their weight and blood pressure, give them the probiotic supplementation, and answer any question about the weight loss program eating plan; under the supervision of a dietary consultant. Collection of the follow-up data for all participated women in order to evaluate the effect of hypocaloric high fiber regimen, physical exercise and probiotic on the improvement of health was performed every month in form of medical advice; to handle any medical complain; under supervision of professor of internal medicine. At the end of the program the following investigation was done which include blood pressure , anthropometric, BMR, laboratory analysis**;** and in addition, After 3 months for MetS criteria and studied Gut microbiota composition changes in form of re-evaluation of *probiotic strains* in the stool:

- Re-collect stool specimens from the same persons and repeat the first step to determine the counts of *Bifidobacterium*, *Lactobacillus*, Firmicutes, Bacteroidetes and *Streptococcus* after supplemented with the probiotic product.
- Evaluation of the effect of probiotic and hypocaloric high fiber regimen reduction bacteria on the improvement of health.

### Statistical analysis

Data were analyzed using the Statistical Package for Social Sciences (SPSS/Windows Version 18, SPSS Inc., Chicago, IL, USA). Normality of data was tested using the Kolmogorov–Smirnov test. The data were normally distributed. So, the parametric tests were used.

All participated women were obese; with BMI ≥ 30 kg/m^2^. They were classified according to the presence of MetS criteria into two subgroups: 35 obese without MetS (have no or less than 2 criteria of MetS), and 23 obese with MetS (have 3 or more criteria of MetS).

The results of the parametric data were expressed as mean ± SE, and mean differences were considered significant at p < 0.05 and highly significant at p < 0.01. The various parametric variables before and after intervention were analyzed and compared using Paired t test. Pearson’s correlation analyses were performed to explore associations between Firmicutes/Bacteroidetes Ratio with different variables and criteria of MetS. Percentages of changes in the criteria of MetS before and after intervention phase were calculated as number and percentage, and drawn using EXCL program.

### Compliance with ethical standards

This research paper was done on Egyptian obese women. It was derived from a cross-sectional survey of a project funded by National Research Centre (NRC) Egypt, 2019–2022 entitled ‘‘Gut Microbiota in Obesity and Metabolic syndrome among obese women: Interactions of the Microbiome, Epigenetic, Nutrition and Probiotic Intervention.” (12th Research Plan of the NRC), which was approved from “Ethics Committee of NRC” (Registration Number is19/236). This study was performed in accordance with the ethical standards as laid down in the 1964 Declaration of Helsinki and its later amendments. A written informed consent was obtained from all participants after being informed about the purpose of the study.

## Results

### Comparisons between the studied two groups before and after intervention

The means ± SE of the blood pressure, the anthropometric measurements and BMR were studied in Table 1. After intervention, data revealed numerical decrease in the SBP of the two groups and significant difference in the DBP of the obese MetS patients group. Body weight and BMI both showed high significant differences in both groups, while the MWC only decreased significantly in the obese MetS patients at p ≤ 0.001. Basal metabolic rate decreased significantly in both groups (Table 1).

**Table 1:** Mean ± SE of the blood pressure, anthropometric measurements and basal metabolic rate among the two studied groups before and after intervention.

Parameters	Obese without MetS (N = 35) Mean ± SE					Obese with MetS (N = 23) Mean ± SE				
	Before		After			Before		After		
	Mean	± SE	Mean	+ SE	P	± SE	Mean	± SE	Mean	P
SBP (mmHg)	112.00	± 1.28	110.00	1.08	0.242	142.94	1.75	138.70	5.38	0.267
DBP (mmHg)	70.00	1.08	69.00	1.37	0.471	90.00	3.08	78.24	1.17	0.000**
Weight (Kg)	89.36	± 2.80	88.44	2.70	0.010*	93.77	2.56	91.21	1.98	0.002**
BMI (kg/m^2^)	37.51	± 0.75	37.11	0.68	0.012*	39.56	0.62	38.54	0.39	0.003**
MWC (CM)	109.20	± 1.73	109.20	2.39	1.000	119.00	3.32	112.50	1.96	0.001**
BMR (KJ/day)	6264.40	144.06	6198.20	135.77	0.001**	6573.35	171.08	6510.61	152.34	0.008*

BMI, body mass index; BMR, basal metabolic rate; MWC, minimal waist circumference; SBP, systolic blood pressure; DBP, diastolic blood pressure.

Significant difference *p ≤ 0.05 **0.01.

Regarding the measured biochemical parameters, the results of the obese without MetS group revealed significant decrease in FBG, insulin concentration and HOMA IR. Obese MetS patients showed significant decrease at p ≤ 0.02–0.000 in the serum concentration of LDL-C, insulin concentration and HOMA-IR. Serum concentration of the triglyceride and total cholesterol showed only numerical decrease, while HDL-C revealed significant increase at p ≤ 000−0.002 in both group (Table 2).

**Table 2:** Mean ± SE of the measured biochemical parameters among the two studied groups before and after intervention.

Parameters	Obese without MetS (N = 35)					Obese with MetS (N = 23)				
	Before		After		P	Before		After		P
	Mean	± SE	Mean	± SE	P	Mean	± SE	Mean	± SE	P
FBG (mg/l)	123.80	9.54	85.40	2.30	0.002**	119.30	3.37	114.35	1.85	0.352
Insulin (mlU/L)	12.38	1.33	7.30	0.63	0.000**	21.22	1.11	11.80	0.87	0.000**
HOMA-IR	4.67	0.86	1.45	0.10	0.000**	6.42	0.49	3.35	0.27	0.000
Lipid profile										
TG (mg/l)	99.40	5.19	98.20	5.51	0.562	162.74	18.92	152.96	12.46	0.242
TC (mg/l)	188.60	6.02	185.60	5.65	0.059	198.65	8.81	191.83	4.96	0.169
HDL-C (mg/l)	50.64	0.98	56.60	1.48	0.000**	52.06	0.78	58.17	2.31	0.002**
LDL-C (mg/l)	112.80	5.25	110.00	4.81	0.160	111.57	2.23	102.52	4.90	0.016*

FBG: Fasting blood glucose, TC: Total Cholesterol, LDL-C: Low density lipoprotein. HDL-P: High density lipoprotein, TG: Triglyceride, Significant difference *p ≤ 0.05 **0.01 .

Concerning the log number and types of microbiota among the two studied groups, data showed that after intervention Log Lactobacillus, Log Bifidobacterium and Log Bacteroidetes increased significantly in both groups, while Log Firmicutes and the Firmicutes/ Bacteroidetes Ratio revealed significant decrease. In the same time data showed significant differences at p ≤ 0.05 between the log number of the four types of bacteria in each group either before or after intervention (Table 3).

**Table 3:** Mean ± SE of the log number and types of Microbiota among the two studied groups before and after intervention.

Type of Microbiota	Obese without MetS (N = 35) Mean ± SE		Obese with MetS(N = 23) Mean ± SE	
	Before	After	Before	After
Log Lactobasillus	6.1096 ± 0.096	6.4724 ± 0.16*a	5.8462 ± 0.174	6.1208 ± 0.19 *b
Log Bifidobacterium	6.1352 ± 0.091	6.8023 ± 0.10*a	6.1200 ± 0.121	6.6412 ± 0.15 *b
Log Bacteroidetes	13.2459 ± 0.196	13.5737 ± 0.18*a	13.0548 ± 0.205	13.2858 ± 0.32 *b
Log Firmicutes	9.4530 ± 0.194	8.2139 ± 0.14**a	8.9127 ± 0.298	8.2448 ± 0.23 *b
P (Between the 4 groups)	0.021	0.030	0.020	0.022
Firmicutes/Bacteroid Ratio	0.7197 ± 0.016	0.6121 ± 0.02*a	0.6890 ± 0.028	0.6334 ± 0.03 *b

Significant difference *p ≤ 0.05 *a obese before vs obese after, *b Obese MetS before vs obese MetS after.

### The daily nutrients intake of the two groups before the dietary intervention and the percent recommended dietary allowance (RDAs)

The results showed high consumption of energy, protein, fat, saturated fat and cholesterol which decreased significantly in the followed dietary regimen. Monounsaturated fatty acids (MUFAs) and poly unsaturated fatty acids (PUFAs) were significantly higher than the followed dietary regimen, but still in the range of the RDAs. Carbohydrates consumption revealed significant decrease in the followed dietary regimen at p ≤ 0.05–0.000 among the obese with and without MetS participant respectively, while the dietary fiber increased in the same way. The daily intake of vitamins A, D, E and C of both groups was below the RDAs before intervention, yet their values showed abundant and significant improvement in the followed dietary regimen. The same effect was observed as regard the daily intake of the mineral calcium (Ca), iron (Fe) and zinc (Zn). Daily consumption of sodium and potassium decreased significantly in the followed dietary regimen (Table 4).

**Table 4:** Mean ± SE and %of the RDAs of nutrients intake before the dietary regimen followed for 3 months of the two studied groups, and the standard RDAs for each category of the nutrient intake.

Nutrient intake	Obese without MetS (N = 35) Mean ± SE %of RDAs	Obese with Mets(N = 23) Mean ± SE %of RDAs	P	Dietary Regimen followed for 3 months Mean ± SE %of RDAs	Standard RDAs
	Before	Before			
Energy (Cal)	2253.45 ± 9.41 102.43	2520.13 ± 8.32, **b 114.55		1074.29 ± 15.34 48.83	2200
Protein (g)	74.76 ± 3.69 149.52	86.04 ± 3.52 , **b 172.08	0.000	44.19 ± 5.90 88.38	50
Fat (g)	105.89 ± 6.39 137.52	120.09 ± 5.73 , **b 155.96	0.007	44.41 ± 2.31 57.67	77
Carbohydrate(g)	250.35 ± 4.51 83.45	273.79 ± 6.10, **b 91.26	0.000	124.46 ± 6.29 41.48	300
Dietary fiber (g)	17.59 ± 0.34*a 70.36	15.86 ± 0.41*b 63.44	0.001	23.76 ± 0.27 95.04	25
Vit. A (µg)	498.65 ± 0.07*a 62.33	476.92 ± 0.13**b 59.62	0.004	792.47 ± 7.53 99.06	800
Vit. D (µg)	2.89 ± 0.09*a 57.80	2.11 ± 0.05**b 42.20	0.005	4.42 ± 0.18 88.40	5
Vit. E(mg)	7.25 ± 1.60*a 48.33	6.32 ± 1.41*b 42.13	0.002	13.40 ± 1.19 89.33	15
Vit. C (mg)	25.47 ± 2.64**a 42.45	27.70 ± 2.71**b 46.17	0.008	42.79 ± 2.03 95.09	60
Sodium (mg)	1506.12 ± 21.11**a 100.41	1510.30 ± 24.01**b 100.69	0.003	875.32 ± 8.51 58.35	1500
Potassium (mg)	1520.80 ± 16.25*a 76.04	1357.19 ± 12.30*b 67.86	0.001	916.91 ± 4.19 45.84	2000
Calcium (mg)	381.06 ± 8.17**a 47.63	338.14 ± 7.11**b 42.27	0.013	761.20 ± 2.90 95.15	800
Iron (mg)	5.22 ± 0.50*a 65.25	4.87 ± 0.30**b 60.88	0.006	7.36 ± 0.77 92.00	8
Zinc (mg)	5.43 ± 1.07*a 45.25	5.10 ± 1.03*b 42.50	0.018	8.60 ± 1.05 71.66	12
Sat. FA (g)	33.71 ± 1.13*a 13.46	37.89 ± 1.09*b 13.53	0.039	9.03 ± 1.10 7.56	No more than 7% of Total Calories intake
MUFs (g)	29.02 ± 0.04*a 11.59	24.07 ± 0.05*b 8.60	0.021	15.72 ± 2.13 13.16	12%-14% of Total Calories intake
PUFAs (g)	18.04 ± 0.05*a 7.20	16.11 ± 0.03*b 5.75	0.042	11.71 ± 1.17 9.81	6%-8% of Total Calories intake
Cholesterol (mg)	238.30 ± 3.17**a 119.15	264.73 ± 6.72**b 132.37	0.051	105.33 ± 5.47 52.66	200

Significant difference *p ≤ 0.05 **0.01. a: obese without MetS before vs Dietary Regimen followed for 3 months, b: Obese with MetS before vs Dietary Regimen followed for 3 months.

### Correlations between Firmicutes/Bacteroidetes Ratio and the criteria of MetS of the studied two groups before and after intervention

In obese without MetS group, positive significant association was detected with LDL-C and non-significant one with DBP before intervention, and with LDL-C and insulin after intervention with p ≤ 0.05. In obese with MetS the significant positive associations were found with DBP, TG, LDL-C and HDL-C, before intervention and with TG, LDL-C and HDL-C after intervention (p ≤ 0.05–0.01). Systolic blood pressure, BMR, anthropometric and biochemical parameters included BMI. MWC, FBG, HOMA-IR and TC all showed negative correlations in which the p values ranged between non-significant to 0.01before and after intervention in both groups, while TG and HDL-C revealed this negative correlations only before intervention in the obese non MetS participants (Table 5).

**Table 5:** Correlation coefficient between firmicutes/bacteroidetes ratio and criteria of Mets of the two studied groups before and after intervention.

Parameters	Firmicutes/bacteroidetes ratio							
	Obese without MetS (N = 35)				Obese with MetS (N = 23)			
	Before		After		Before		After	
	R	P	R	p	R	P	R	P
SBP	− 0.037	0.832	− 0.407	0.015*	− 0.231	0.288	− 0.230	0.292
DBP	0.269	0.118	− 0.250	0.148	0.668	0.000**	− 0.237	0.276
BMI	− 0.316	0.064	− 0.223	0.199	− 0.906	0.000**	− 0.961	0.000**
MWC	− 0.032	0.854	− 0.145	0.406	− 0.800	0.000**	− 0.809	0.000**
BMR	− 0.696	0.000**	− 0.552	0.001**	− 0.438	0.036*	− 1.000	0.000**
FBG	− 0.394	0.019*	− 0.219	0.207	− 0.877	0.000**	− 0.904	0.000**
Insulin	− 0.497	0.002**	0.371	0.028*	− 0.370	0.082	0.284	0.190
HOMA-IR	− 0.882	0.000**	− 0.823	0.000**	− 0.827	0.000**	− 0.479	0.021*
TC	− 0.647	0.000**	− 0.920	0.000**	− 0.637	0.001**	− 0.411	0.051
TG	− 0.441	0.008**	− 0.781	0.000**	0.695	0.000**	0.870	0.000**
HDL-C	− 0.867	0.000**	− 0.887	0.000**	0.991	0.000**	0.916	0.000**
LDL-C	0.369	0.029*	0.343	0.044*	0.819	0.000**	0.444	0.034*

Significant difference *p ≤ 0,05 **0.01.

### Frequency distribution of the metabolic syndrome criteria before and after intervention among the two studied groups

**Figure 1:**
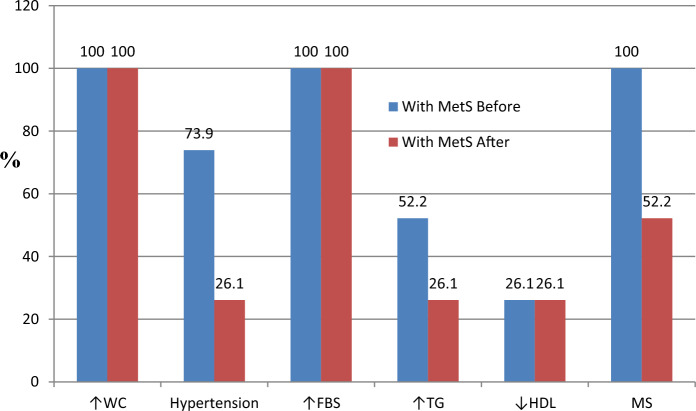
Frequency distribution of the metabolic syndrome criteria before and after intervention among obese women with MetS.

Table 6 revealed that out of the 23 obese women had MetS before intervention, 11 patients improved and had 2 criteria only after intervention, while 12 women (52.2%) only still had the syndrome. The percent of improvement was mainly in the blood pressure (26.1% after 73.9%) and TG (26.1% after 52.2%), while there were no changes in the percentages of patients had high MWC and FBG and low HDL. While those without MetS the improvement was mainly in FBG (0% after 20%). Figure 1 Also demonstrate that after the intervention there was improvement in the criteria of MetS among patients with MetS, particularly the blood pressure and the serum concentration of triglycerides .

**Table 6:** Frequency distribution of the metabolic syndrome criteria before and after intervention among the two studied groups.

	Obese without MetS (N = 35)				Obese with MetS (N = 23)			
	Before		After		Before		After	
	N	%	N	%	N	%	N	%
High MWC	35	100	35	100	23	100	23	100
Hypertension	0	0	0	0	17	73.9	6	26.1
High FBS	7	20.0	0	0	23	100	23	100
HighTG	0	0	0	0	12	52.2	6	26.1
Low HDL	14	40	14	40	6	26.1	6	26.1
MetS criteria								
1 criteria	14	24.1	21	60.0				
2 criteria	21	36.2	14	40.0				
3 criteria					11	47.8	6	26.1
4 criteria					12	52.2	6	26.1
MetS	0	0	0	0	23	100	12	52.2

## Discussion

Metabolic syndrome is defined as the accumulation of impaired glucose tolerance, dyslipidemia, hypertension, and central obesity with insulin resistance^15^. Based on this definition, the results of this survey showed that more than one quarter (39.7%) of the obese women shared in this study were suffering from metabolic syndrome, and this was evident from the reported anthropological and biochemical measurements of the participants.

The role of the intestinal microbiota in metabolic disorders is increasingly being considered**.** Although the microbiota is influenced by various factors, diet seems to be the main contributor to its diversity^16^. All the type of diet and its calorie content are able to change the relative proportion of intestinal microbes (increase in Firmicutes with a simultaneous decrease in Bacteroides) and consequently their ability to obtain energy from food^17^. Also Proença et al.^18^ said that changes in gut microbiota have been linked to both obesity and metabolic syndrome. With regard to the bacterial groups involved, a comparison of the intestinal microbiota of obese and slim people showed a higher proportion of Firmicutes and a lower proportion of Bacteroidetes in obese people^19^.

It was clear from current study that the values of the calorie consumed derived from fat and carbohydrates were high among each of the obese female participants, as well as those with metabolic syndrome compared to RDAs. This was accompanied by an increase in the number of gut bacteria, namely bacteroidetes and firmicutes, compared to other studied beneficial bacteria. The results revealed abundance of Bacteroidetes bacteria over the Firmicutes with low Firmicutes/Bacteroidetes ratio. This results were in agreement with Patil et al.^20^ who explicit that bacteroidetes was distinguished among obese subjects and its abundance absolutely correlative with BMI. In addition the composition of bacteria living in the different parts of the intestine is variable per age, body weight, geologic site, as well as diet^21^.

### Intervention

The GI tract is home to over 1014 microorganisms, with approximately 10 times more bacterial cells than human cells and 100 times more genomic content (Microbiome) than the human genome^22^. However, a recently revised estimate suggests that the ratio of human to bacterial cells is closer to 1:1^23^.

Probiotics as live microorganisms will confer health advantages to the host once administered in adequate amounts. There's considerable proof of their nutritional and immunological disorder properties together with reports that elucidate the association of probiotics with the F/B ratio, fatness, and inflammatory bowel disease (IBD). Administered probiotics orally can contribute to the restoration of dysbiotic microbiota and to the improvement of obesity or IBD^8^.

After sufficient time of intervention with the probiotics and the hypocaloric high fiber diet in addition to doing light exercise, the findings obtained proved a reduction in the weight of the patients which accompanied by a decrease in the other anthropometric measurements, including the BMI and waist circumference. It also showed an improvement in all the biochemical parameters characteristic of the metabolic syndrome, in addition to reduction in the diastolic blood pressure and decreases BMR.

In this regard, Wang et al.^24^ noted that during the last forty years, the incidence of obesity which is strongly linked to the gut microbiota and chronic inflammation has rapidly increased. As a result, probiotic-based modification of the gut microbiota composition has been developed as a treatment for obesity.

Yang et al.^25^ stated that the increased recorded blood pressure in the spontaneously hypertensive rats was associated with the Firmicutes: Bacteroidetes ratio, these changes are accompanied by a decrease in the microbial population that produces acetic acid and butyric acid. The microbial abundance, diversity, and uniformity of the gut microbiota were associated with significantly reduction of blood pressure values. In this regard, the various bacteria administered (Lactobacillus, Bifidobacterium, Streptococcus thermophiles) convert food ingredients into active metabolites that have a positive effect on immune cell function. Decreased inflammatory immune cell function may promote blood pressure lowering effects^26^. The results of these experiment and research are in complete agreement with the recorded decrease in blood pressure that was recorded in our patients.

Follow-up study for many years of individuals sharing in an extreme weight loss program found that all patients had regained virtually all of their pre-program weight, that especially due to decrease of the basal metabolic rate (BMR) that is known as Resting Metabolic Rate (RMR) adaptation^27^. In this case, the gut microbiota was become an environmental factor that regulates the host’s energy balance. It increases the host's ability to recover energy from digested food and to produce metabolites and microbial products such as short-chain fatty acids, secondary bile acids, and lipopolysaccharides. These metabolites and microbial products act as signaling molecules that regulate appetite, intestinal motility, energy absorption and storage, and energy expenditure^28^.

Probiotics and / or prebiotics are promising for improving insulin sensitivity by actively altering the composition of intestinal microbial communities, lowering intestinal endotoxin levels^29^. In addition, Cronin et al.^6^ said that consumption of fibers virtually reverses the characteristic changes in metabolic parameters associated with obesity and MetS through microbial fermentation followed by the production of short-chain fatty acids (SCFAs) that improves lipids profile and glucose in patients had diseases resulting in metabolic disorders.

Numerous human studies have demonstrated increased the Firmicutes/Bacteroidetes (F/B) proportion in obese people compared to lean people, and tended to decrease with weight loss^8,19,30^, while other studies have produced conflicting results^31,32^. In this respect, data of the present study showed that Log Lactobacillus, Log Bifidobacterium and Log Bacteroidetes increased significantly in both groups, while Log Firmicutes and the Firmicutes/Bacteroidetes Ratio revealed significant decrease after intervention. The Firmicutes/Bacteroidetes (F/B) ratio is widely accepted to have an important influence in maintaining normal intestinal homeostasis. Increased or decreased F/B ratio is regarded as dysbiosis, whereby the former is usually observed with obesity, and the latter with IBD.

The important correlation coefficient between the Firmicutes / Bacteroidetes ratio and the MetS criteria revealed positive significant correlation between the ratio and the serum LDL-C in both groups, and the serum triglyceride concentration in the obese MetS group, before and after intervention, and to the DBP before intervention. Meanwhile the negative correlation reported between the ratio and the other metabolic syndrome criteria despite of the reduction found in their means values could be attributed to the presence of intragroup variations and unequal response of the parameters to the dietary therapy that was followed by the participants. In addition valuable data regarding the effects of certain probiotics do not guarantee sustained amelioration of insulin resistance in humans^33^.

The overall, findings of the current study showed pronounce beneficial effect of using probiotics with a hypocaloric diet rich in fiber on all criteria of the metabolic syndrome. In addition, the results showed a decrease in diastolic blood pressure and the triglycerides concentration among a significant proportion of the patients.

In conclusion, the results of this study highlight a positive trend of probiotics supplementation with hypocaloric high fiber diet and the light exercise on the improvement and amelioration of the anthropometric parameters of obese Egyptian women with the related metabolic diseases. Hence the putative mechanisms of probiotic and prebiotic action develop dietary strategies for the management of metabolic syndrome. In this respect, the focus of the future research should be to evaluate the efficacy of different probiotic strains, the quantities to be administered, and the duration of the intervention.

## Data Availability

The datasets used and/or analyzed during the current study are available from the corresponding author on reasonable request, after taking the permission of our institute “National Research Centre”.
